# Longitudinal trend in post-discharge estimated glomerular filtration rate in intensive care survivors

**DOI:** 10.1177/17511437241308673

**Published:** 2024-12-26

**Authors:** Rebecca M Glendell, Kathryn A Puxty, Martin Shaw, Malcolm AB Sim, Jamie P Traynor, Patrick B Mark, Mark Andonovic

**Affiliations:** 1 Undergraduate Medical School, College of Medical, Veterinary and Life Sciences, University of Glasgow, Glasgow, UK; 2 Department of Intensive Care Medicine, Glasgow Royal Infirmary, Glasgow, UK; 3 Department of Anaesthesia, Critical Care and Pain, School of Medicine, University of Glasgow, Glasgow, UK; 4 Department of Intensive Care, Queen Elizabeth University Hospital, Glasgow, UK; 5 Glasgow Renal and Transplant Unit, Queen Elizabeth University Hospital, Glasgow, UK; 6 School of Cardiovascular and Metabolic Health, University of Glasgow, Glasgow, UK

**Keywords:** Acute kidney injury, intensive care units, glomerular filtration rate, risk factors, long term adverse effects

## Abstract

**Background:**

Acute kidney injury (AKI) within the intensive care unit (ICU) is common but evidence is limited on longer-term renal outcomes. We aimed to model the trend of kidney function in ICU survivors using estimated glomerular filtration rate (eGFR), comparing those with and without AKI, and investigate potential risk factors associated with eGFR decline.

**Methods:**

This observational cohort study included all patients aged 16 or older admitted to two general adult ICUs in Scotland between 1st July 2015 and 30th June 2018 who survived to 30 days following hospital discharge. Baseline serum creatinine and subsequent values were used to identify patients with AKI and calculate eGFR following hospital discharge. Mixed effects modelling was used to control for repeated measures and to allow inclusion of several exploratory variables.

**Results:**

3649 patients were included, with 1252 (34%) experiencing in-ICU AKI. Patients were followed up for up to 2000 days with a median 21 eGFR measurements. eGFR declined at a rate of −1.9 ml/min/1.73m^2^/year (*p-*value < 0.001) in the overall ICU survivor cohort. Patients with AKI experienced an accelerated rate of post-ICU eGFR decline of −2.0 ml/min/1.73m^2^/year compared to a rate of −1.83 ml/min/1.73m^2^/year in patients who did not experience AKI (*p-*value 0.007). Pre-existing diabetes or liver disease and in-ICU vasopressor support were associated with accelerated eGFR decline regardless of AKI experience.

**Conclusions:**

ICU survivors experienced a decline in kidney function beyond that which would be expected regardless of in-ICU AKI. Long-term follow-up is warranted in ICU survivors to monitor kidney function and reduce morbidity and mortality.

## Introduction

Acute kidney injury (AKI) incidence is high in patients with critical illness, at approximately 40%–60%, compared to 10%–15% of the general hospital population.^1–4^ Survival following critical illness is improving but many patients develop long-term morbidity requiring increased healthcare resources following discharge.^5^

Following AKI, patients have been demonstrated to be at increased risk of chronic kidney disease (CKD) and worsening of pre-existing CKD, and this risk increases with AKI severity.^6–10^

Conventionally, acquisition of long-term renal outcomes for analysis requires studying patients until they develop kidney failure or require dialysis or a kidney transplant. This requires the study of a large cohort as kidney failure events are relatively infrequent, even in high-risk populations.^11^ However, eGFR slopes have been verified as a viable surrogate for clinical endpoints in randomised control trials and are a validated tool in assessment of long-term kidney function.^12–14^ Emerging evidence supports the use of repeated data measurements, particularly using eGFR as a marker of kidney function, to inform long-term monitoring and follow-up in ICU survivors.^15^

Renal injury incurred during ICU may lead to longer-term renal dysfunction, however, evidence is limited. A study by Haines et al. found a rapid initial decline in post-discharge eGFR in ICU survivors followed by a slower decline up to 7 years follow-up.^15^ Consequently, the authors advocated for a post-ICU follow-up clinic to assess long-term kidney function and provide intervention to reduce morbidity and mortality.^15^ Although the National Institute for Health and Care Excellence (NICE) recommended in their 2023 update that patients who experience in-hospital AKI should have a clinical review within 3 months or sooner if they are deemed to be high risk,^16^ historically, actual follow-up rates have varied and were generally low.^17–21^ As ICU survivors may present a group at higher risk of poor kidney outcomes in both the short and longer term, it may be more appropriate to identify and prioritise patients at the highest risk of accelerated longitudinal decline in kidney function. Furthermore, precise predictors of outcomes following ICU-associated AKI, as well as post-ICU discharge care, have been identified as areas for further study in this emerging field.^22^

This study sought to model the trend of post-ICU discharge kidney function in ICU survivors; to compare post-discharge kidney function in patients who were diagnosed with in-ICU AKI to those who were not; and to investigate potential risk factors associated with kidney function decline.

## Methods

This observational cohort study used retrospective analysis of an existing dataset of routinely gathered prospectively acquired data from patients’ ICU admission and subsequent follow-up.^4^ Ethics approval was granted by the NHS Health Research Authority, London-Surrey Research Ethics Committee (REC reference: 18/LO/2060).

### Patient population

All patients aged 16 or older admitted to two large Scottish general adult ICUs (Glasgow Royal Infirmary and the Queen Elizabeth University Hospital, serving approximately 1.1 million people) between 1st July 2015 and 30th June 2018 were identified. The study population consisted of ICU survivors, determined as patients alive at 30 days following hospital discharge to avoid patients discharged for palliative care from influencing results. Exclusion criteria included long-term kidney replacement therapy (KRT) or prior kidney transplantation. Sample size was determined by number of patients identified over the pre-determined study period.

### Data collection

Patients were identified using the Scottish Intensive Care Society Audit Group (SICSAG) Wardwatcher™ database which has capture of all patients admitted to ICU. Patient identifiers were then input into the Strathclyde Electronic Renal Patient Record (SERPR) database (VitalPulse, UK). SERPR has a record of all patients receiving long-term KRT across the West of Scotland as well as active interfaces that automatically retrieve data from laboratory, radiology and death records. This includes historical laboratory data from 01/01/2009 onwards, ensuring important outcomes were available for this study. Full details are described in previously published work on the same patient cohort.^4^ Patients without available eGFR data following discharge were considered lost to follow-up and excluded from all analyses.

Follow-up data were retrieved from routinely gathered eGFR measurements at subsequent hospital or general practice appointments. eGFR measurements from the 30 days following hospital discharge were not included to mitigate any acute effects of illness and ICU on kidney function. However, it should be noted that the reason for collection of the eGFR measurements is unknown, so although measurements within 30 days of discharge were not included, subsequent measurements could have been taken at a point of acute illness and eGFR therefore may not have stabilised.

Baseline demographic variables including age, sex, admitting specialty, APACHE II score, admission diagnosis and type of organ support were retrieved from the SICSAG database. Admitting specialty was categorised as medical or surgical. Admission diagnoses were organised into groups according to coding in the SICSAG database and diagnoses such as sepsis were grouped regardless of causative pathogen. Organ support was categorised based on receipt of invasive mechanical ventilation, vasopressor support (infusion of vasoactive medication including vasopressors or inotropes) and KRT. Pre-existing comorbidities were identified using data in ICU electronic patient records and grouped according to cardiovascular disease (including chronic hypertension and ischaemic heart disease), respiratory disease (including obstructive airway disease and interstitial lung disease), liver disease (including alcoholic and non-alcoholic fatty liver disease, hepatitis or cirrhosis) diabetes mellitus (of all types) and cancer (all types and stages).

### AKI determination

Pre-admission serum creatinine results were used to calculate a baseline value for each patient. Either the median value from 8 to 365 days before admission, or, if these data were unavailable, the lowest value in the week prior to admission was used. Patients without data available for classification of baseline kidney function were excluded. This serum creatinine value was used to calculate baseline eGFR using the CKD-EPI equation.^23^ This reference value was then used to diagnose AKI during ICU admission using Kidney Disease Improving Global Outcomes (KDIGO) 2012 classification.^21^ The initial injury was identified as the point at which AKI criteria were met for the first time and severity of AKI was classified using the highest creatinine value from the duration of injury. Patients who received KRT whilst in ICU were automatically classified as having AKI stage 3. AKI was classified using only serum creatinine values due to a lack of urine output data. Patients who were readmitted to ICU during the study period were not re-evaluated for AKI on their second or any subsequent admissions.

### Statistical analysis

Statistical analysis was conducted using the statistical software R (The R Foundation; R version 4.2.2).

For baseline demographics, continuous variables were summarised using median values and interquartile range (IQR) and compared using Wilcoxon rank sum test. Categorical variables were summarised using proportions with 95% confidence intervals (95%CI) and compared using Pearson’s Chi-squared test.

Mixed effects modelling was used throughout determination of eGFR trend to control for repeated measures and missing data, and to allow inclusion of several exploratory variables. The dataset was capped at a maximum follow-up of 2000 days due to increased variability in eGFR measurements after this due to significantly fewer patients having follow-up data available after this time point.

When investigating potential risk factors associated with kidney function decline, the complete dataset was divided into two cohorts: patients who experienced in-ICU AKI and those who did not. Initial unadjusted models were built for each variable in each cohort and univariable *p*-values < 0.1 were included in the multivariable adjusted models. Two adjusted models for each cohort were created: one investigating the effect of pre-ICU variables and one investigating the effect of in-ICU variables on longitudinal kidney function. Multivariable *p-*values < 0.05 were regarded as statistically significant.

## Results

**Figure 1.**
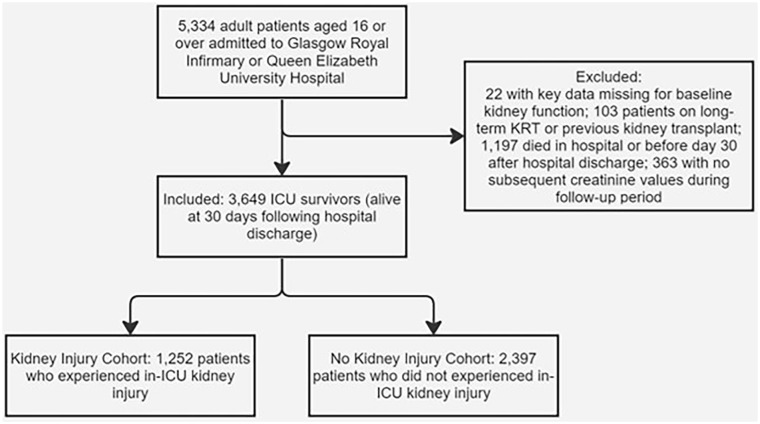
Generation of the final cohort included in analysis. Graphical representation of the final cohort generation from all patients who met the inclusion criteria to splitting of the final cohort into those who experienced kidney injury in ICU and those who did not.

3649 patients met the inclusion criteria (Figure 1) with 134,038 eGFR measurements (median 21 per patient). The minimum and maximum follow up times were 6 days and 2000 days respectively (median 1229 days). Main causes for ICU admission were sepsis (20.0%), malignancy (13.0%), gastrointestinal disease (excluding perforation; 8.7%) and trauma (8.1%). Of the 3649 patients, 1252 (34.3%) experienced AKI whilst in ICU. Patients who experienced AKI were older (median age of 60 compared to 56); had a higher APACHE II score (19 compared to 12); a lower baseline eGFR (83 ml/min/1.73m^2^ compared to 95 ml/min/1.73m^2^); a lower calculated eGFR on discharge (78 ml/min/1.73m^2^ compared to 102 ml/min/1.73m^2^); and there was a higher proportion with a past medical history (PMH) of cardiovascular disease, liver disease and diabetes (Table 1).

**Table 1.** Baseline patient demographics grouped according to kidney injury in ICU.

Characteristic	Overall, N = 3649^a^	Injury, N = 1252^a^	No kidney injury, N = 2,397^a^	*p*-Value^b^
Sex				<0.001
Female	1696 (46%)	514 (41%)	1182 (49%)	
Male	1953 (54%)	738 (59%)	1215 (51%)	
Age	57 (44, 69)	60 (48, 71)	56 (42, 68)	<0.001
Baseline eGFR	92 (73, 106)	83 (62, 99)	95 (80, 109)	<0.001
eGFR at ICU discharge^c^	96 (76, 111)	78 (41, 101)	102 (88, 114)	<0.001
Unknown	78 (2.1%)	10 (0.8%)	68 (2.8%)	
Admitting Specialty				<0.001
Medical	1326 (36%)	518 (41%)	808 (34%)	
Surgical	2323 (64%)	734 (59%)	1589 (66%)	
Patients who underwent surgery	1735 (48%)	470 (38%)	1265 (53%)	<0.001
Elective	794 (22%)	122 (10%)	672 (28%)	
Emergency	941 (26%)	348 (28%)	593 (25%)	
Sepsis	722 (20%)	375 (30%)	347 (14%)	<0.001
APACHE II score	14 (10, 20)	19 (15, 24)	12 (9, 17)	<0.001
Ventilation support	1780 (49%)	820 (65%)	960 (40%)	<0.001
Ventilation support days^d^	0.0 (0.0, 2.0)	2.0 (0.0, 5.0)	0.0 (0.0, 2.0)	<0.001
Kidney Replacement Therapy	238 (6.5%)	238 (19%)	0 (0%)	<0.001
Kidney Replacement Therapy days^d^	0.00 (0.00, 0.00)	0.00 (0.00, 0.00)	0.00 (0.00, 0.00)	<0.001
Vasopressor support	1517 (42%)	831 (66%)	686 (29%)	<0.001
Vasopressor support days^d^	2.0 (1.0, 4.0)	2.0 (2.0, 5.0)	2.0 (1.0, 3.0)	<0.001
Cardiovascular history	1377 (38%)	550 (44%)	827 (35%)	<0.001
Respiratory history	732 (20%)	253 (20%)	479 (20%)	0.9
Diabetes history	509 (14%)	245 (20%)	264 (11%)	<0.001
Liver history	308 (8.4%)	122 (9.7%)	186 (7.8%)	0.041
Cancer history	286 (7.8%)	87 (6.9%)	199 (8.3%)	0.15

Table showing baseline patient demographics for the whole cohort and grouped according to kidney injury in ICU.

a Median (IQR); n (%).

b Wilcoxon rank sum test; Pearson’s Chi-squared test.

c Value at discharge or up to 1 week after.

d In patients who received respective organ support.

In the cohort of ICU survivors with AKI, 37%, 20% and 43% of patients experienced AKI stages 1, 2 and 3 respectively.

### Renal function in ICU survivors

Initial linear mixed effects modelling of all ICU survivors identified a decline in eGFR over 2000 days at a rate of −1.9 ml/min/1.73m^2^/year (*p-*value < 0.001). After adjustment for age, a decline in eGFR was observed at a rate of −1.28 ml/min/1.73m^2^/year (*p*-value < 0.001; Table 2).

**Table 2.** Summary of models built to explore eGFR rate of decline and level in the whole patient cohort and with interaction for kidney injury.

Interactions	Predicted annual rate of eGFR decline from baseline up to 2000 days post-ICU discharge (ml/min/1.73m^2^; 95%CI)	*p-*Value
Complete study cohort	−1.90 (−1.96, −1.84)	<0.001
With adjustment for age	−1.28 (−1.50, −1.06)	<0.001
Interaction for kidney injury		0.007
Kidney injury	−2.00 (−2.10, −1.91)	
No kidney injury	−1.83 (−1.95, −1.70)	
Interaction for kidney injury with adjustment for age		0.0497
Kidney injury	−1.39 (−1.64, −1.14)	
No kidney injury	−1.26 (−1.39, −1.14)	
	Difference in baseline eGFR level with kidney injury compared to no kidney injury (ml/min/1.73m^2^)	*p-*Value
Interaction for kidney injury	−14.61 (−16.36, −12.87)	<0.001
Interaction for kidney injury (with adjustment for age)	−10.96 (−12.32, −9.6)	<0.001

Table showing model output exploring eGFR decline in the whole patient cohort and with interaction for kidney injury.

**Figure 2.**
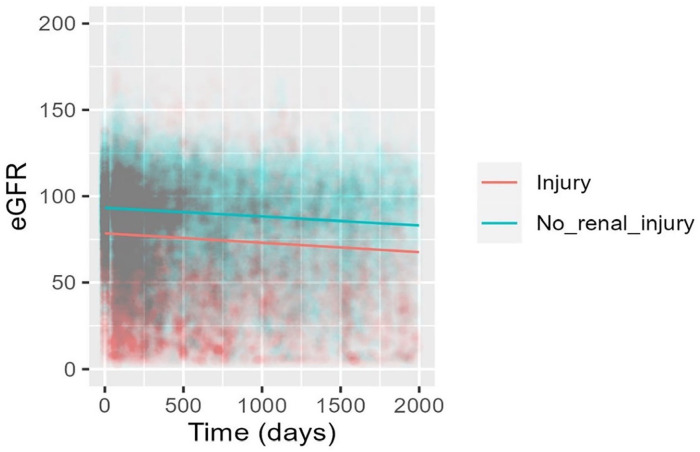
Predicted eGFR by in-ICU kidney injury, adjusted for age (years). Graph showing eGFR decline over days in the kidney injury cohort compared to the no kidney injury cohort with the slope in the no kidney injury cohort starting at a higher baseline eGFR but declining at a similar rate to the kidney injury cohort.

eGFR declined at a rate of −2.00 ml/min/1.73m^2^/year in patients who experienced in-ICU AKI, compared to −1.83 ml/min/1.73m^2^/year in patients who did not (*p-*value = 0.007 for interaction; Table 2). Comparison of the two eGFR slopes can be viewed in Figure 2.

After adjustment for age, the eGFR slope declined at a rate of −1.39 ml/min/1.73m^2^/year in patients who experienced AKI compared to −1.26 ml/min/1.73m^2^/year in patients who did not (*p*-value = 0.0497).

### Identifying risk factors in patients without AKI

Factors associated with accelerated eGFR decline in patients without AKI in ICU are summarised in Table 3 and Supplemental Table 1.

**Table 3.** Results of univariable and multivariable modelling for association between in-ICU variables and post-discharge eGFR results in patients who did not experience kidney injury in ICU.

Variable	Univariable change in baseline eGFR (ml/min/1.73m^2^) (95%CI)	*p-*Value	Multivariable change in baseline eGFR (ml/min/1.73m^2^) (95%CI)	*p-*Value
Length of stay in ICU (days)	0.20 (−0.02, 0.42)	0.08	0.17 (−0.46, 0.80)	0.60
Length of ventilation support (days)	0.75 (0.48, 1.03)	<0.001	0.85 (0.16, 1.54)	0.02
Length of vasopressor support (days)	0.004 (−0.58, 5.86)	0.99	−1.12 (−1.91, −0.32)	0.006
Specialty (surgical)	−8.45 (−10.46, −6.44)	<0.001	−2.68 (−6.52, 1.17)	0.17
Sepsis	1.40 (−1.33, 4.14)	0.31	-	-
Interactions	Univariable change in eGFR rate (ml/min/1.73m^2^/year) (95%CI)	*p-*Value	Multivariable change in eGFR rate (ml/min/1.73m^2^/year) (95%CI)	*p-*Value
Length of stay in ICU (days)	−0.04 (−0.06, −0.019)	<0.001	−0.02 (−0.08, 0.038)	0.50
Length of ventilation support (days)	−0.02 (−0.04, 0.003)	0.10	0.15 (0.09, 0.22)	<0.001
Length of vasopressor support (days)	−0.08 (−0.14, −0.029)	0.002	−0.2 (−0.27, −0.13)	<0.001
Specialty (surgical)	0.13 (−0.03, 0.29)	0.11	-	-
Sepsis	0.015 (−0.19, 0.22)	0.88	-	-

Table showing model output for in-ICU variables and post-discharge eGFR results in patients who did not experience kidney injury in ICU with univariable and multivariable change in baseline eGFR and eGFR rate of decline.

#### Pre-ICU variables

Pre-ICU variables associated with an increased rate of eGFR decline following ICU discharge included pre-existing diabetes (−0.53 ml/min/1.73m^2^/year, *p-*value < 0.001) and pre-existing liver disease (−2.30 ml/min/1.73m^2^/year, *p*-value < 0.001). A history of respiratory disease was associated with a reduced decline in eGFR following ICU discharge of 0.27 ml/min/1.73m^2^/year (*p-*value = 0.004).

#### In-ICU variables

Length of vasopressor support was associated with an accelerated annual rate of eGFR decline of −0.20 ml/min/1.73m^2^/year per day of vasopressor support (*p-*value < 0.001). Length of ventilation support was associated with a reduced annual rate of decline in eGFR by 0.15 ml/min/1.73m^2^/year per day of support (*p-*value < 0.001).

### Identifying risk factors in patients with AKI

Results of the univariable and multivariable models for patients with AKI are summarised in Supplemental Tables 2 and 3.

#### Pre-ICU variables

Pre-existing conditions associated with an increased annual rate of eGFR decline were: cardiovascular disease (−0.64 ml/min/1.73m^2^/year, *p-*value < 0.001); liver disease (−0.68 ml/min/1.73m^2^/year, *p-*value < 0.001); diabetes (−1.75 ml/min/1.73m^2^/year, *p-*value < 0.001); and cancer (−0.75 ml/min/1.73m^2^/year, *p-*value = 0.001).

A history of respiratory disease was associated with a reduced annual rate of eGFR decline of 0.39 ml/min/1.73m^2^/year (*p-*value = 0.004).

#### In-ICU variables

AKI stage was associated with an increased annual rate of eGFR decline of −1.21 ml/min/1.73m^2^/year for stage 2 and −1.85 ml/min/1.73m^2^/year for stage 3 compared to stage 1 (*p-*value < 0.001 for both).

With regards to organ support, length of ventilation support was associated with a reduced rate of eGFR decline by 0.09 ml/min/1.73m^2^/year per day of support. Length of receipt of KRT was also associated with a reduced rate of eGFR decline by 0.11 ml/min/1.73m^2^/year per day of support (*p-*value < 0.001 for both). There was also a significant reduction in baseline eGFR of −0.50 ml/min/1.73m^2^ per day of KRT (*p-*value = 0.04).

Length of vasopressor support was associated with an accelerated annual rate of decline in eGFR by −0.06ml/min/1.73m^2^/year (*p-*value < 0.001) per day of support.

Finally, sepsis was associated with a reduced annual rate of eGFR decline of 1.54 ml/min/1.73m^2^ (*p-*value < 0.001).

## Discussion

This study of ICU survivors demonstrated an annual decline in eGFR beyond what would be expected due to age alone.^24–28^ While previous studies have suggested the annual decline in eGFR would be expected to be between 0.6–0.9 ml/min/1.73m^2^ due to a year’s increase in age,^24–28^ ICU survivors experienced a decline of −1.9 ml/min/1.73m^2^/year. Anticipating that some of this decline may be age-related, age adjusted analysis demonstrated eGFR decline in ICU survivors of −1.28 ml/min/1.73m^2^, a difference of 0.62 ml/min/1.73m^2^, consistent with expected age-related decline. These results are consistent with the findings of Haines et al. that ICU survivors experience continued decline in kidney function in the years following ICU discharge.^15^

AKI incidence in our study was found to be 34.3% which is lower than the 40%–60% in-ICU AKI incidence stated in literature.^1–4^ Although critical care populations are heterogenous, the lower incidence found in our study is likely due to exclusion of patients who did not survive up to 30 days following ICU discharge from the study population. As AKI is associated with high mortality, it is likely that in-ICU AKI incidence in our cohort was higher.

In contrast to the findings of Haines et al. who noted renal decline to be similar in patients who experienced in-ICU AKI to those who did not, this study observed an accelerated longitudinal rate of eGFR decline in ICU survivors who experienced in-ICU AKI compared to those who did not.^15^ However, although the difference in eGFR decline between the two cohorts was statistically significant, an increased annual rate of decline of −0.13 ml/min/1.73m^2^ (accounting for age) in patients who experienced AKI compared to those who did not is not clinically significant and falls below the rate of eGFR slope decline which is considered significant in CKD trials.^12^ More relevant perhaps, is the finding that ICU survivors experienced an accelerated rate of eGFR decline in the years following discharge, above that which is attributable to ageing alone, regardless of in-ICU AKI experience. This suggests that ICU survivors are at risk of accelerated decline in kidney function warranting dedicated follow-up regardless of AKI experience in ICU.

This study also explored factors associated with accelerated kidney function decline. Existing factors associated with accelerated eGFR decline following ICU-discharge in all patients included a past medical history of diabetes or liver disease. Diabetes mellitus is a known risk factor for CKD and patients with diabetes are more likely to develop end stage kidney disease (ESKD).^29^ The association between liver and kidney disease has also long been known, with hepatorenal syndrome a clinical example of this,^30^ and a recent meta-analysis found that non-alcoholic fatty liver disease (NAFLD) was associated with a moderately increased risk of CKD stage⩾ 3, according to KDIGO classification.^31^ Therefore, it is unsurprising that we found pre-existing diabetes or liver disease to be associated with accelerated decline in post-ICU kidney function.

A history of malignancy was associated with an accelerated rate of eGFR decline in the patient cohort who experienced in-ICU AKI. This could be a result of nephrotoxic chemotherapeutic agents used in some cancer treatment making the kidneys susceptible to greater damage following in-ICU injury.^32,33^ However, not all patients with cancer will have received chemotherapy or nephrotoxic treatment so further research is required before drawing more definitive conclusions.

Cardiovascular disease was not associated with any changes in eGFR slope in the no AKI cohort, however, was associated with an increased rate of decline in the AKI cohort. Cardiovascular and kidney disease are known to be linked, with cardiovascular factors including hypertension increasing CKD risk.^34,35^ As such, the fact that there was no association in the no AKI cohort is surprising. Liver and kidney disease share many cardiovascular and metabolic risk factors meaning these comorbidities may account for some of the expected association seen in cardiovascular disease.^35,36^

The fact that pre-existing respiratory disease was associated with a decreased rate of eGFR decline in both cohorts is surprising and there is no current physiological explanation for this. The statistically significant difference in eGFR is very small however, and likely not clinically relevant. A possible explanation for this finding is that the statistical significance could be a random observation.

This study also explored factors related to ICU stay on subsequent kidney function. Increasing length of vasopressor support was associated with an increased rate of eGFR decline. This association could be as great as an accelerated decline of −1.4 ml/min/1.73m^2^/year in a patient who did not experience AKI and received a week of vasopressor support. This is highly clinically significant and identifies a group of ICU survivors at potentially greater risk of long-term accelerated kidney function decline.

Vasopressors are routinely used in ICU in the management of conditions such as vasodilatory shock, following cardiovascular surgery and after myocardial infarction.^37^ Adverse effects of vasopressors include excessive vasoconstriction which can result in organ under-perfusion, with the kidneys being particularly vulnerable due to their dense microvasculature.^37^ Increasing duration of vasopressor support could therefore increase the risk of kidney under-perfusion and subsequent damage, resulting in accelerated eGFR decline.

In patients who experienced AKI, length of receipt of KRT was associated with a reduced rate of eGFR decline. This is contrary to findings by Soum et al. who found increasing length of receipt of KRT to be a risk factor for developing severe, long-term CKD in critically ill patients requiring KRT for AKI treatment.^38^ One explanation for our findings is that there was a significant difference in baseline eGFR of −0.50 ml/min/1.73m^2^ per day of KRT suggesting that patients who require KRT in ICU have a lower pre-ICU baseline eGFR. Therefore, this could result in a relative reduction in eGFR decline compared to patients with a higher baseline, explaining the observed protective effect.

Length of ventilatory support was associated with a reduced rate of eGFR decline in both cohorts. One possible explanation for this could be that muscle wasting as a result of increased duration of ventilatory support could have resulted in lower serum creatinine measurements and therefore overestimation of eGFR resulting in a reduced rate of eGFR decline.

An in-ICU diagnosis of sepsis was associated with a reduced rate of eGFR decline in patients who experienced AKI, although the difference was negligible. Again, this is surprising as sepsis is a common cause of AKI and is associated with renal vasoconstriction and reduced oxygenation which can impair kidney function.^39,40^ Sepsis is associated with significant morbidity and mortality,^40^ therefore, this observed association could be explained by survivor bias with surviving patients more likely to have been in better health prior to ICU admission and therefore suffering from less consequent kidney damage.

### Implications of our findings

Our finding of accelerated eGFR decline in ICU-survivors concurs with the conclusion made by Haines et al. that long term follow-up of renal function, potentially through community eGFR monitoring, is warranted in ICU-survivors to reduce the morbidity and mortality associated with CKD.^15^ However, follow-up of all ICU-survivors may not be feasible.^17,18^ Therefore ICU-survivors should be risk stratified according to pre-existing comorbidities and length of vasopressor support to prioritise those at greatest risk.

### Strengths and limitations

A strength of this study was that it utilised a large dataset likely sufficiently powered for the statistical analyses undertaken. Patients included were from two centres with a mixed general ICU population and can likely be considered representative of the UK critical care population.^15^ However, AKI is a syndrome with high heterogeneity with regards to causes and not all phenotypes will follow the same disease pattern. Furthermore, the use of mixed effects modelling accounted for repeated measures, missing values and several exploratory variables.

Limitations of this study include those associated with retrospective study design and these results should be viewed as exploratory rather than explanatory. Furthermore, the eGFR equation is not validated in patients under the age of 18, but due to patients aged 16–17 being occasionally admitted to the general adult ICUs investigated in this study, 19 patients in this age group were included. However, these patients are likely to exhibit adult physiology and as only 19 patients were included this is unlikely to have influenced results. Patients who were dialysis dependent on hospital discharge were also included. eGFR calculation is not representative of kidney function in those who are dialysis dependent, however, due to the relatively small number of patients in this group (n = 17), these eGFR measurements are also unlikely to have influenced results.

A lack of urine output data to determine in-ICU AKI likely resulted in underestimation of AKI incidence. Furthermore, inclusion of patients without serum creatinine readings prior to the week before admission and using lowest serum creatinine in the week before admission might have resulted in inaccuracies as this value could have already be influenced by illness and therefore not represent a true baseline.

The CKD-EPI equation used to calculate eGFR solely used serum creatinine as a measure of kidney function. Serum creatinine is influenced by factors including muscle mass which can fall during ICU admission resulting in overestimation of kidney function.^41^ This could have particularly affected the AKI patient group due to AKI being associated with increased morbidity and therefore likely increased muscle wasting, as well as any patients who underwent limb amputation. Therefore, patients in these groups could have experienced greater longitudinal decline in kidney function than our results show. Although, muscle mass is usually regained to some extent following ICU discharge so therefore effects could be smaller in those with longer follow up. Future studies could utilise Cystatin C measurement as a biomarker for kidney function as it is not associated with muscle mass, to greater reflect kidney function in these patient groups.^41^ Alternatively, proteinuria could be used as a marker of kidney damage and predictor of CKD progression.

eGFR follow-up data were gathered from routinely collected measurements. As follow-up of kidney function tends to occur more often in patients with worse renal function,^17^ this introduces potential bias. Moreover, due to difficulties with model convergence, two final multivariable models were built for each patient cohort. This means that any interaction between pre-ICU and in-ICU variables hasn’t been accounted for in the two separate models. Finally, as many of the variables investigated are inherently linked, potential confounding makes interpretation of results more uncertain.

## Conclusion

This study found that ICU survivors experienced accelerated decline in kidney function represented by eGFR slopes. Whilst patients with in-ICU AKI suffered an accelerated rate of eGFR decline compared to those without in-ICU AKI, all patients suffered accelerated rate of eGFR decline compared to that expected by age alone. Pre-existing comorbidities, AKI stage and increasing length of vasopressor support were associated with accelerated eGFR decline. Long-term follow-up is warranted in high-risk ICU survivors to monitor kidney function and reduce morbidity and mortality associated with CKD. Further work is required to help risk stratify ICU survivors at highest risk of accelerated decline in kidney function and determine feasibility of more widespread follow-up of kidney function.

## Supplemental Material

sj-docx-1-inc-10.1177_17511437241308673 – Supplemental material for Longitudinal trend in post-discharge estimated glomerular filtration rate in intensive care survivors Supplemental material, sj-docx-1-inc-10.1177_17511437241308673 for Longitudinal trend in post-discharge estimated glomerular filtration rate in intensive care survivors by Rebecca M Glendell, Kathryn A Puxty, Martin Shaw, Malcolm AB Sim, Jamie P Traynor, Patrick B Mark and Mark Andonovic in Journal of the Intensive Care Society

## References

[1] Ronco C Bellomo R Kellum JA. Acute kidney injury. Lancet 2019; 394: 1949–1964. 31777389 10.1016/S0140-6736(19)32563-2

[bibr2-17511437241308673] Mo S Bjelland TW Nilsen TIL , et al. Acute kidney injury in intensive care patients: incidence, time course, and risk factors. Acta Anaesthesiol Scand 2022; 66: 961–968. 35674748 10.1111/aas.14100 PMC9543500

[bibr3-17511437241308673] Hoste EAJ Bagshaw SM Bellomo R , et al. Epidemiology of acute kidney injury in critically ill patients: the multinational AKI-EPI study. Intensive Care Med 2015; 41: 1411–1423. 26162677 10.1007/s00134-015-3934-7

[4] Andonovic M Traynor JP Shaw M , et al. Short- and long-term outcomes of intensive care patients with acute kidney disease. EClinicalMedicine 2022; 44: 101291. 35198917 10.1016/j.eclinm.2022.101291 PMC8850318

[5] Lone NI Gillies MA Haddow C , et al. Five-year mortality and hospital costs associated with surviving intensive care. Am J Respir Crit Care Med 2016; 194: 198–208. 26815887 10.1164/rccm.201511-2234OC PMC5003217

[6] Chawla LS Kimmel PL. Acute kidney injury and chronic kidney disease: an integrated clinical syndrome. Kidney Int 2012; 82: 516–524. 22673882 10.1038/ki.2012.208

[bibr7-17511437241308673] Chawla LS Eggers PW Star RA , et al. Acute kidney injury and chronic kidney disease as interconnected syndromes. New Engl J Med 2014; 371: 58–66. 24988558 10.1056/NEJMra1214243 PMC9720902

[bibr8-17511437241308673] Forni LG Darmon M Ostermann M , et al. Renal recovery after acute kidney injury. Intensive Care Med 2017; 43: 855–866. 28466146 10.1007/s00134-017-4809-x PMC5487594

[bibr9-17511437241308673] Chawla LS Amdur RL Amodeo S , et al. The severity of acute kidney injury predicts progression to chronic kidney disease. Kidney Int 2011; 79: 1361–1369. 21430640 10.1038/ki.2011.42 PMC3257034

[10] Coca SG Singanamala S Parikh CR. Chronic kidney disease after acute kidney injury: a systematic review and meta-analysis. Kidney Int 2012; 81: 442–448. 22113526 10.1038/ki.2011.379 PMC3788581

[11] Keith DS Nichols GA Gullion CM , et al. Longitudinal follow-up and outcomes among a population with chronic kidney disease in a large managed care organization. Arch Intern Med 2004; 164: 659–663. 15037495 10.1001/archinte.164.6.659

[12] Inker LA Heerspink HJL Tighiouart H , et al. GFR slope as a surrogate end point for kidney disease progression in clinical trials: a meta-analysis of treatment effects of randomized controlled trials. J Am Soc Nephrol 2019; 30: 1735–1745. 31292197 10.1681/ASN.2019010007 PMC6727261

[bibr13-17511437241308673] Pasala S Carmody JB. How to use. . . serum creatinine, cystatin C and GFR. Arch Dis Child Educ Pract Ed 2017; 102: 37–43. 10.1136/archdischild-2016-311062 27647862

[14] Inker LA Collier W Greene T , et al. A meta-analysis of GFR slope as a surrogate endpoint for kidney failure. Nat Med 2023; 29: 1867–1876. 37330614 10.1038/s41591-023-02418-0 PMC13037386

[15] Haines RW Powell-Tuck J Leonard H , et al. Long-term kidney function of patients discharged from hospital after an intensive care admission: observational cohort study. Sci Rep 2021; 11: 9928. 33976354 10.1038/s41598-021-89454-3 PMC8113423

[16] Quality statement 6: Clinical review after hospital discharge|Acute kidney injury|Quality standards|NICE, https://www.nice.org.uk/guidance/qs76/chapter/Quality-statement-6-Clinical-review-after-hospital-discharge (accessed 11 November 2024).

[17] Choon XY Lumlertgul N Cameron L , et al. Discharge documentation and follow-up of critically ill patients with acute kidney injury treated with kidney replacement therapy: a retrospective cohort study. Front Med 2021; 8: 710228. 10.3389/fmed.2021.710228 PMC8476795 34595187

[bibr18-17511437241308673] Lumlertgul N Wright R Hutson G , et al. Long-term outcomes in patients who received veno-venous extracorporeal membrane oxygenation and renal replacement therapy: a retrospective cohort study. Ann Intensive Care 2022; 12: 70. 35870022 10.1186/s13613-022-01046-0 PMC9308118

[bibr19-17511437241308673] Scenario: management of acute kidney injury | Management | Acute kidney injury | CKS | NICE, https://cks.nice.org.uk/topics/acute-kidney-injury/management/management-of-acute-kidney-injury/ (accessed 12 July 2023).

[bibr20-17511437241308673] Clinical practice guideline acute kidney injury (AKI), www.nice.org.uk/accreditation (accessed 13 July 2023).

[21] KDIGO clinical practice guideline for acute kidney injury. DOI: 10.1038/kisup.2012.1.

[22] Vijayan A Abdel-Rahman EM Liu KD , et al. Recovery after critical illness and acute kidney injury. Clin J Am Soc Nephrol 2021; 16: 1601–1609. 34462285 10.2215/CJN.19601220 PMC8499012

[23] Levey AS Stevens LA Schmid CH , et al. A new equation to estimate glomerular filtration rate. Ann Intern Med 2009; 150: 604–612. 19414839 10.7326/0003-4819-150-9-200905050-00006 PMC2763564

[24] Glassock RJ Rule AD. Aging and the kidneys: anatomy, physiology and consequences for defining chronic kidney disease. Nephron 2016; 134: 25–29. 27050529 10.1159/000445450

[bibr25-17511437241308673] Glassock RJ Winearls C. Ageing and the glomerular filtration rate: truths and consequences. Trans Am Clin Climatol Assoc 2009; 120: 419–428. 19768194 PMC2744545

[bibr26-17511437241308673] Denic A Glassock RJ Rule AD. Structural and functional changes with the ging kKidney. Adv Chronic Kidney Dis 2016; 23: 19–28. 26709059 10.1053/j.ackd.2015.08.004 PMC4693148

[bibr27-17511437241308673] Gallet M Drouet C Berriolo-Riedinger A , et al. Effect of obesity, age and gender on glomerular filtration rate measured in normal adults. Ann Biol Clin 2021; 79: 57–61. 10.1684/abc.2021.1615 33527907

[28] Lindeman RD Tobin J Shock NW. Longitudinal studies on the rate of decline in renal function with age. J Am Geriatr Soc 1985; 33: 278–285. 3989190 10.1111/j.1532-5415.1985.tb07117.x

[29] Shen Y Cai R Sun J , et al. Diabetes mellitus as a risk factor for incident chronic kidney disease and end-stage renal disease in women compared with men: a systematic review and meta-analysis. Endocrine 2017; 55: 66–76. 27477292 10.1007/s12020-016-1014-6

[30] Gupta K Bhurwal A Law C , et al. Acute kidney injury and hepatorenal syndrome in cirrhosis. World J Gastroenterol 2021; 27: 3984–4003. 34326609 10.3748/wjg.v27.i26.3984 PMC8311533

[31] Mantovani A Petracca G Beatrice G , et al. Non-alcoholic fatty liver disease and risk of incident chronic kidney disease: an updated meta-analysis. Gut 2022; 71: 156–162. 33303564 10.1136/gutjnl-2020-323082

[32] Xiao Z Jiang Y Chen XF , et al. The hepatorenal toxicity and tumor response of chemotherapy with or without aidi injection in advanced lung cancer: a meta-analysis of 80 randomized controlled trials. Clin Ther 2020; 42: 515–543.e31. 10.1016/j.clinthera.2020.01.011 32088021

[33] Singh AK Hussain S Ahmed R , et al. Impact of imatinib treatment on renal function in chronic myeloid leukaemia patients. Nephrology 2022; 27: 318–326. 34894374 10.1111/nep.14014

[34] Major RW Cheng MRI Grant RA , et al. Cardiovascular disease risk factors in chronic kidney disease: a systematic review and meta-analysis. PLoS One 2018; 13: e0192895. 10.1371/journal.pone.0192895 PMC5862400 29561894

[35] Byrne CD Targher G. NAFLD as a driver of chronic kidney disease. J Hepatol 2020; 72: 785–801. 32059982 10.1016/j.jhep.2020.01.013

[36] Chang YT Wu JL Hsu CC , et al. Diabetes and end-stage renal disease synergistically contribute to increased incidence of cardiovascular events: a nationwide follow-up study during 1998–2009. Diabetes Care 2014; 37: 277–285. 23920086 10.2337/dc13-0781

[37] Russell JA Gordon AC Williams MD , et al. Vasopressor therapy in the intensive care unit. Semin Respir Crit Care Med 2021; 42: 59–77. 32820475 10.1055/s-0040-1710320

[38] Soum E Timsit JF Ruckly S , et al. Predictive factors for severe long-term chronic kidney disease after acute kidney injury requiring renal replacement therapy in critically ill patients: an ancillary study of the ELVIS randomized controlled trial. Crit Care 2022; 26: 367. 36447221 10.1186/s13054-022-04233-4 PMC9706988

[39] Skytte Larsson J Krumbholz V Enskog A , et al. Renal blood flow, glomerular filtration rate, and renal oxygenation in early clinical septic shock. Crit Care Med 2018; 46: e560–e566. 10.1097/CCM.0000000000003088 29517549

[40] Peerapornratana S Manrique-Caballero CL Gómez H , et al. Acute kidney injury from sepsis: current concepts, epidemiology, pathophysiology, prevention and treatment. Kidney Int 2019; 96: 1083–1099. 31443997 10.1016/j.kint.2019.05.026 PMC6920048

[41] Ferguson TW Komenda P Tangri N. Cystatin C as a biomarker for estimating glomerular filtration rate. Curr Opin Nephrol Hypertens 2015; 24: 295–300. 26066476 10.1097/MNH.0000000000000115

