# Supplementary material for: Longitudinal trend in post-discharge estimated glomerular filtration rate in intensive care survivors
Source: J Intensive Care Soc. 2024 Dec 26;26(1):29–37. doi: 10.1177/17511437241308673 (PMC11670225; doi:10.1177/17511437241308673)
Supplement: sj-docx-1-inc-10.1177_17511437241308673 – Supplemental material for Longitudinal trend in post-discharge estimated glomerular filtration rate in intensive care survivors [file sj-docx-1-inc-10.1177_17511437241308673.docx]

**Longitudinal trend in post-discharge estimated Glomerular Filtration Rate in intensive care survivors- Supplementary Material 1**

Rebecca M Glendell^1^, Kathryn A Puxty^2,3^, Martin Shaw^3^, Malcolm AB Sim^3,4^, Jamie P Traynor^5^, Patrick B Mark^5,6^, Mark Andonovic^3^

1. University of Glasgow, Undergraduate Medical School, College of Medical, Veterinary and Life Sciences, Glasgow, UK
2. Department of Intensive Care Medicine, Glasgow Royal Infirmary, Glasgow, UK
3. Department of Anaesthesia, Critical Care and Pain, School of Medicine, University of Glasgow, Glasgow, UK
4. Department of Intensive Care, Queen Elizabeth University Hospital, Glasgow, UK
5. Glasgow Renal and Transplant Unit, Queen Elizabeth University Hospital, Glasgow, UK
6. School of Cardiovascular and Metabolic Health, University of Glasgow, Glasgow, UK

Correspondence: [2433165g@student.gla.ac.uk](mailto:2433165g@student.gla.ac.uk)

Table 1: Results of univariable and multivariable modelling for association between pre-ICU variables and post-discharge eGFR results in patients who did not experience kidney injury in ICU……………………………………………………………2

Table 2: Results of univariable and multivariable modelling for association between pre-ICU variables and post-discharge eGFR results in patients who experienced kidney injury in ICU…………………………………………………………………………3

Table 3: Results of univariable and multivariable modelling for association between in-ICU variables and post-discharge eGFR results in patients who experienced kidney injury in ICU…………………………………………………………………………4

Table 1: Results of univariable and multivariable modelling for association between pre-ICU variables and post-discharge eGFR results in patients who did not experience kidney injury in ICU.

| **Variable** | **Univariable change in baseline eGFR (ml/min/1.73m^2^) (95%CI)** | ***p-*value** | **Multivariable change in baseline eGFR (ml/min/1.73m^2^) (95%CI)** | ***p-*value** |
| --- | --- | --- | --- | --- |
| Age (years) | -0.91 (-0.95, -0.87) | <0.001 | -0.86 (-0.91, -0.81) | <0.001 |
| Sex (Male) | 6.80 (4.89, 8.71) | <0.001 | 4.03 (2.61, 5.46) | <0.001 |
| Cardiovascular PMH | -16.35 (-18.26, -14.46) | <0.001 | -1.80 (-3.46, -1.35) | 0.03 |
| Diabetes PMH | -12.55 (-15.57, -9.55) | <0.001 | -4.30 (-6.61, -2.05) | <0.001 |
| Liver PMH | 11.14 (7.56, 14.72) | <0.001 | 4.80 (2.13, 7.39) | <0.001 |
| Respiratory PMH | -2.85 (-5.26, -0.45) | 0.02 | 0.48 (-1.28, 2.24) | 0.60 |
| Cancer PMH | -9.50 (-12.95, -6.05) | <0.001 | -0.86 (-3.38, 1.65) | 0.50 |
| **Interactions** | **Univariable change in eGFR rate (ml/min/1.73m^2^/year) (95%CI)** | ***p-*value** | **Multivariable change in eGFR rate (ml/min/1.73m^2^/year) (95%CI)** | ***p-*value** |
| Age (years) | -0.02 (-0.02, -0.01) | <0.001 | -0.02 (-0.024, -0.014) | <0.001 |
| Sex (Male) | -0.23 (-0.38, -0.08) | 0.003 | -0.15 (-0.30, 0.006) | 0.06 |
| Cardiovascular PMH | -0.28 (-0.44, -0.12) | <0.001 | 0.014 (-0.16, 0.19) | 0.87 |
| Diabetes PMH | -0.655 (-0.87, -0.44) | <0.001 | -0.53 (-0.76, -0.31) | <0.001 |
| Liver PMH | -2.25 (-2.51, -1.98) | <0.001 | -2.3 (-2.57, -2.04) | <0.001 |
| Respiratory PMH | 0.35 (0.17, 0.53) | <0.001 | 0.27 (0.08, 0.45) | 0.004 |
| Cancer PMH | -0.23 (-0.52, 0.05) | 0.12 | - | - |

Alt text: Table showing model output for pre-ICU variables and post-discharge eGFR results in patients who did not experience kidney injury in ICU with univariable and multivariable change in baseline eGFR and eGFR rate of decline.

Table 2: Results of univariable and multivariable modelling for association between pre-ICU variables and post-discharge eGFR results in patients who experienced kidney injury in ICU.

| **Variable** | **Univariable change in baseline eGFR (ml/min/1.73m^2^) (95%CI)** | ***p-*value** | **Multivariable change in baseline eGFR (ml/min/1.73m^2^) (95%CI)** | ***p-*value** |
| --- | --- | --- | --- | --- |
| Age (years) | -0.99 (-1.07, -0.91) | <0.001 | -0.93 (-1.02, -8.39) | <0.001 |
| Sex (Male) | 5.93 (2.78, 9.07) | <0.001 | - | - |
| Cardiovascular PMH | -16.67 (-19.65, -13.69) | <0.001 | -3.87 (-6.69, -1.04) | 0.007 |
| Diabetes PMH | -8.39 (-12.26, -4.53) | <0.001 | -5.38 (-8.61, -2.14) | 0.001 |
| Liver PMH | 8.71 (3.49, 13.92) | 0.001 | 1.39 (-2.91, 5.70) | 0.53 |
| Respiratory PMH | -4.75 (-8.62, -0.88) | 0.02 | 0.47 (-2.72, 3.67) | 0.77 |
| Cancer PMH | -5.55 (-11.64, 0.55) | 0.07 | 1.51 (-3.51, 6.54) | 0.55 |
| **Interactions** | **Univariable change in eGFR rate (ml/min/1.73m^2^/year) (95%CI)** | ***p-*value** | **Multivariable change in eGFR rate (ml/min/1.73m^2^/year) (95%CI)** | ***p-*value** |
| Age (years) | -0.002 (-0.009, 0.004) | 0.50 | 0.004 (-0.004, 0.01) | 0.31 |
| Sex (Male) | -0.13 (-0.34, 0.07) | 0.20 | - | - |
| Cardiovascular PMH | -0.58 (-0.78, -0.38) | <0.001 | -0.64 (-0.88, -0.41) | <0.001 |
| Diabetes PMH | -1.80 (-2.05, -1.57) | <0.001 | -1.75 (-1.98, -1.51) | <0.001 |
| Liver PMH | -0.88 (-1.17, -0.59) | <0.001 | -0.68 (-0.97, -0.39) | <0.001 |
| Respiratory PMH | 0.40 (0.13, 0.66) | 0.003 | 0.39 (0.13, 0.66) | 0.004 |
| Cancer PMH | -0.39 (-0.83, 0.06) | 0.09 | -0.75 (-1.20, -0.30) | 0.001 |

Alt text: Table showing model output for pre-ICU variables and post-discharge eGFR results in patients who experienced kidney injury in ICU with univariable and multivariable change in baseline eGFR and eGFR rate of decline.

Table 3: Results of univariable and multivariable modelling for association between in-ICU variables and post-discharge eGFR results in patients who experienced kidney injury in ICU.

| **Variable** | **Univariable change in baseline eGFR (ml/min/1.73m^2^) (95%CI)** | ***p-*value** | **Multivariable change in baseline eGFR (ml/min/1.73m^2^) (95%CI)** | ***p-*value** |
| --- | --- | --- | --- | --- |
| Length of stay in ICU (days) | 0.16 (0.00004, 0.31) | 0.05 | 0.029 (-0.44, 0.49) | 0.90 |
| AKI stage |  |  |  |  |
| Stage 1 |  |  |  |  |
| Stage 2 | -4.30 (-8.45, -0.14) | 0.04 | -3.49 (-8.50, 1.51) | 0.17 |
| Stage 3 | -15.40 (-18.74, -12.03) | <0.001 | -13.29 (-17.56, -9.01) | <0.001 |
| Length of ventilation support (days) | 0.24 (0.04, 0.44) | 0.02 | 0.31 (-0.23, 0.86) | 0.26 |
| Length of vasopressor support (days) | 0.27 (0.002, 0.52) | 0.04 | 0.20 (-0.25, 0.65) | 0.38 |
| Length of renal support (days) | -0.7 (-1.12, -0.28) | 0.001 | -0.50 (-0.98, -0.01) | 0.045 |
| Specialty (surgical) | -0.8 (-3.96, -7.67) | 0.62 | -2.29 (-6.02, 1.46) | 0.23 |
| Sepsis | -4.11 (-7.50, -0.71) | 0.02 | -3.17 (-6.87, 0.51) | 0.09 |
| **Interactions** | **Univariable change in eGFR rate (ml/min/1.73m^2^/year) (95%CI)** | ***p-*value** | **Multivariable change in eGFR rate (ml/min/1.73m^2^/year) (95%CI)** | ***p-*value** |
| Length of stay in ICU (days) | 0.04 (0.03, 0.05) | <0.001 | -0.02 (-0.05, 0.02) | 0.40 |
| AKI stage |  |  |  |  |
| Stage 1 |  |  |  |  |
| Stage 2 | -1.08 (-1.39, -0.77) | <0.001 | -1.21 (-1.59, -0.83) | <0.001 |
| Stage 3 | -1.33 (-1.57, -1.08) | <0.001 | -1.85 (-2.16, -1.54) | <0.001 |
| Length of ventilation support (days) | 0.05 (0.04, 0.07) | <0.001 | 0.09 (0.05, 0.13) | <0.001 |
| Length of vasopressor support (days) | 0.05 (0.03, 0.07) | <0.001 | -0.06 (-0.09, -0.03) | <0.001 |
| Length of renal support (days) | 0.12 (0.09, 0.15) | <0.001 | 0.11 (0.08, 0.15) | <0.001 |
| Specialty (surgical) | -0.23 (-0.44, -0.03) | 0.026 | 0.23 (-0.03, 0.49) | 0.09 |
| Sepsis | 1.03 (0.81, 1.26) | <0.001 | 1.54 (1.30, 1.80) | <0.001 |

Alt text: Table showing model output for in-ICU variables and post-discharge eGFR results in patients who experienced kidney injury in ICU with univariable and multivariable change in baseline eGFR and eGFR rate of decline.
